# Dietary protein restriction increases hepatic leptin receptor mRNA and plasma soluble leptin receptor in male rodents

**DOI:** 10.1371/journal.pone.0219603

**Published:** 2019-07-15

**Authors:** Riho Yamada, Shizuka Odamaki, Masaya Araki, Tasuku Watanabe, Keigo Matsuo, Kaito Uchida, Taku Kato, Yori Ozaki-Masuzawa, Asako Takenaka

**Affiliations:** Department of Agricultural Chemistry, School of Agriculture, Meiji University, Kawasaki, Kanagawa, Japan; National Institute for Agronomic Research, FRANCE

## Abstract

Leptin is an adipokine that regulates adipose tissue mass through membrane-anchored leptin receptor (Ob-R). Extracellular domain of Ob-R in plasma is called soluble leptin receptor (sOb-R), and is the main leptin-binding protein. Based on a previous DNA microarray analysis that showed induction of hepatic *Ob-R* mRNA in low-protein diet-fed mice, this study aimed to clarify the effect of dietary protein restriction on hepatic *Ob-R* mRNA and plasma sOb-R levels. First, the effect of protein restriction on hepatic Ob-R mRNA level was examined together with fasting and food restriction using male rats as common experimental model for nutritional research. Hepatic *Ob-R* mRNA level was increased by feeding low-protein diet for 7 d, although not significantly influenced by 12-h fasting and sixty percent restriction in food consumption. Then, effect of protein restriction on liver Ob-R and plasma sOb-R was investigated using male mice because specific sOb-R ELISA was more available for mice. Hepatic *Ob-R* mRNA level was also increased in protein restricted-mice although it did not increase in hypothalamus. Hepatic Ob-R protein was decreased, whereas plasma sOb-R was increased by protein restriction. Because the concentration of sOb-R increased without changing plasma leptin concentration, free leptin in plasma was significantly reduced. The direct effect of amino acid deprivation on *Ob-R* mRNA level was not observed in rat hepatoma cells H4IIE cultured in amino acid deprived medium. In conclusion, dietary protein restriction increased hepatic *Ob-R* mRNA, resulting in increased plasma sOb-R concentration, which in turn, reduces plasma free leptin level and may modulate leptin activity.

## Introduction

Leptin was discovered as a satiety factor, predominantly secreted from adipose tissues and known to maintain adequate fat reserve [1, 2]. Plasma concentration of leptin has been reported to be dependent on body fat mass [2] and studies with leptin-injected mice demonstrated that leptin could reduce appetite, fat mass, and increase energy expenditure [3–5]. In addition to its role in regulating food intake and body fat mass, important roles in glucose homeostasis have also been observed in leptin-injected animals [3–6].

Leptin exerts its effects by binding to its specific receptor, leptin receptor (Ob-R) that has one trans-membrane domain and resembles the gp130 subunit of the interleukin-6-receptor-complex, a member of class I cytokine receptor family [7]. At least 6 Ob-R isoforms, named Ob-Ra–Ob-Rf, have been described and are products of alternative mRNA splicing from a single gene [8, 9]. Among these isoforms, only Ob-Rb has complete length, containing all the motifs required for signal transduction and can fully activate the janus kinase/signal transducers and activators of transcription (JAK/STAT) intracellular signaling pathway [10]. Other Ob-Rs are classified as short forms (Ob-Ra, c, d and f) or secreted form (Ob-Re), the latter having only the extracellular domain and released into plasma [11]. Although short isoforms cannot activate JAK/STAT signaling, they have been demonstrated to activate mitogen-activated protein kinase pathways [12]. Leptin reduces appetite via Ob-Rb in hypothalamus by suppressing the expression and secretion of neuropeptide Y and agouti-related peptide, and enhancing the synthesis of pro-opiomelanocortin [10]. In addition, it reduces energy expenditure by activating sympathetic nervous system [13]. Peripheral tissues have been reported to express mRNA for short isoforms of Ob-R [14, 15], which may exert central nervous system-independent effect of leptin [15].

Ob-Re, also called soluble leptin receptor (sOb-R), is produced by proteolytic cleavage of membrane-anchored Ob-R by a desintegrin and metalloprotease (ADAM) 10 and ADAM17, as well as translation from *Ob-Re* mRNA [16]. Plasma sOb-R can bind to leptin with its ligand-binding domain and is the main leptin-binding protein in human plasma [17]. sOb-R is considered to prevent plasma leptin from binding to cell surface Ob-R, thereby reducing leptin activity [18]. The ratio of freely circulating leptin to sOb-R-bound leptin (plasma leptin/plasma sOb-R) is referred to as free leptin index (FLI) and used as an index of leptin resistance [19]. On the other hand, effect of sOb-R to stabilize and store leptin in plasma has also been reported earlier [20].

Regarding the regulation of sOb-R generation, clinical studies have demonstrated that plasma sOb-R level is low in obese subjects and high in lean subjects, indicating the inverse correlation with plasma leptin concentration and body mass index (BMI) [21–23]. In addition, plasma sOb-R level is increased, depending on the stage of fibrosis, in obese patients with non-alcoholic steatosis, demonstrating its correlation with liver disease [24]. In animal studies, hepatic *Ob-R* mRNA expression and plasma sOb-R level were enhanced by food restriction and 24-h fasting in normal mice and liver-specific insulin receptor knock-out mice [25, 26]. Up-regulation of sOb-R by fasting was also reported in humans [27]. Taken together, the results demonstrated the possibility that sOb-R is increased under conditions of energy shortage; however, precise regulatory mechanisms of sOb-R production remain unclear.

In our previous study investigating the effect of dietary protein restriction on lipid metabolism [28], we performed DNA microarray analysis using livers of rats fed low-protein diet and found that hepatic *Ob-R* mRNA was highly induced by protein deficiency. Therefore, in this study, we investigated the effect of dietary protein restriction on mRNA and protein levels of Ob-R in both liver and plasma and aimed to clarify the regulation of leptin activity by protein nutrition.

## Materials and methods

### Animals

Male Wistar rats were purchased from Japan Laboratory Animals Inc. (Tokyo, Japan) and male C57BL/6J mice were purchased from Japan SLC (Hamamatsu, Japan). The animals were housed in stainless wire cage with a 12-h light:dark cycle (06:00–18:00) at a temperature of 22–24 ^o^C. They were given free access to water and commercial diet (MF, Oriental Yeast Co. Ltd, Japan) to acclimatize to the housing condition. Thereafter, they were fed control diet with 20% casein as nitrogen source (20C, Table 1) for 3 days to acclimatize to the purified powdery diet. During nutrition experiments, they were given 20C or low-protein diet with 5% casein (5C, Table 1) and water. After feeding on experimental diet, the animals were dissected under anesthesia with sodium pentobarbital (64.8 mg/kg intraperitoneally, Somnopentyl; Kyoritsu Seiyaku Co. Ltd, Japan) and tissue (liver, hypothalamus) and blood from heart were collected. Heparinized plasma and tissue samples were immediately frozen in liquid nitrogen and stored at -80 ^o^C until use. This study was carried out in strict accordance with the recommendations in the Guide for the Care and Use of Laboratory Animals of the National Institutes of Health. All animal experiments were approved by the Meiji University Institutional Animal Care and Use Committee (Approval Number: IACUC 15–0007). All surgery was performed under sodium pentobarbital anesthesia, and all efforts were made to minimize suffering.

**Table 1:** Composition of the experimental diet.

	20C	5C
Casein	200	50
α-Cornstarch	434.5	536.1
Sucrose	217.3	268.1
Cellulose	50	50
Mineral mixture (AIN93G)*	35	35
Vitamin mixture (AIN93)*	10	10
Corn oil	50	50
L-Met	3.2	0.8

(g/kg diet)

* The mineral and vitamin mixture were obtained from Oriental Yeast Co., Tokyo, Japan.

### Experiment with rats fed protein-restricted diet in fasting/re-feeding conditions

Five-week-old rats were fed 20C or 5C *ad libitum* for 7 d. Half of the rats in each group were dissected after 12-h fasting and the other half was dissected after 12-h re-feeding the same diet, following the 12-h fasting. Experimental groups were 20C-fasted (20CF, n = 5), 20C-refed (20CR, n = 5), 5C-fasted (5CF, n = 5), and 5C-refed (5CR, n = 5). Liver and heparinized plasma were obtained and stored as described above.

### Experiment with rats under protein restriction or food restriction

Four-week-old rats were divided into three groups and fed 20C *ad libitum* (20C, n = 6), fed 5C *ad libitum* (5C, n = 6), or pair-fed 20C with 5C (20R, n = 6) for 16 d. At the time of dissection, saline was perfused systemically from left ventricle of the heart and released from the right atrium to remove blood from tissues. Liver and heparinized plasma were obtained and stored as described above.

### Experiment with mice fed protein-restricted diet

Five-week-old C57BL/6J mice were fed 20C or 5C *ad libitum* (n = 5 each) for 7 d and dissected after 13-h fasting. At the time of dissection, saline was perfused as described above. Liver, hypothalamus, and heparinized plasma were collected and stored as described above.

### Cell culture

H4IIE-C3 cells (rat hepatoma cell line, ATCC CRL-1600) from the American Tissue and Culture Collection (ATCC) were grown in Dulbecco’s modified Eagle’s medium (DMEM) supplemented with 10% fetal bovine serum (FBS) and antibiotics (Antibiotic-Antimycotic, Thermo Fisher Scientific Inc.) under 5% CO_2_ at 37 ^o^C. At sub-confluency, medium was changed to experimental medium with or without amino acids (1AA or 0AA, respectively; Table 2), and cultured for another 6 h (n = 6 for each medium).

**Table 2:** Experimental media.

	1AA	0AA
10xEarl's salt solution*	50mL	
10xMEM EAA*	10mL	
10xMEM NEAA*	5mL	
20mM L-glutamic acid*	5mL	
100xvitamin mixture*	5mL	5mL
NaHCO_3_*	1.1g	1.1g
100xantibiotic antimycotic solution**	5mL	5mL
bovine serum albumin	0.5g	0.5g

(per 500mL)

*, purchased from SIGMA

**, purchased from Hyclone

### Total RNA extraction and real-time PCR

Total RNA extraction from liver, hypothalamus, and H4IIE cells, cDNA synthesis, and real-time PCR were performed with TriPure Isolation Reagent (Roche Applied Science), PrimeScript RT reagent Kit with gDNA Eraser (Perfect Real Time), and THUNDERBIRD SYBR qPCR Mix (Toyobo), respectively, according to the manufacturers’ instructions as described previously [29]. *β-actin* or *hypoxanthine phosphoribosyltransferase (HPRT)* was used as internal control. Amplification of a single PCR product for each primer set was confirmed with melting curve analysis. Each result was divided by the average value of the control group and expressed as relative mRNA levels. Primers for *Ob-R* were located in extracellular region, and could amplify all Ob-R isoforms. Messenger RNA of *C/EBP homologous protein (CHOP)* was measured in H4IIE cells as as an amino acid-regulated gene. Primer sequences are shown in Table 3.

**Table 3:** Primers for realtime PCR.

gene		sequence(5'-3')
β-actin (rat)	forward	GGCCAACCGTGAAAAGATGA
	reverse	AGAGGCATACAGGGACAACACA
Hprt (rat)*	forward	TGACACTGGCAAAACAATGCA
	reverse	GGTCCTTTTCACCAGCAAGCT
Leptin receptor (rat)	forward	AGTGGGAAGCACTGTGCAGTT
	reverse	GAGCTCTGATGTAGGACGAATAGATG
Chop (rat)**	forward	CGGAACCTGAGGAGAGAGTGTT
	reverse	AATTGGACCGGTTTCTGCTTT
β-actin (mouse)	forward	AAGTGTGACGTTGACATCCGTAA
	reverse	GCAATGCCTGGGTACATGGT
Leptin receptor (mouse)	forward	GGTCCAGGTGAGGAGCAAGA
	reverse	AAAGAAGCATTCGATCCAACACTA
Transmemrane leptin receptor (mouse)	forward	TGGAAGGAGTTGGAAAACCAA
	reverse	TACAGCCCTGCGTCATTCTG

*Hprt, hypoxanthine phosphoribosyltransferase

**Chop, C/EBP homologous protein

### Western blotting

Protein extraction from liver was performed as described previously, except for the use of ultrasonic homogenizer (NR-50M, Microtec Co., Ltd.) to homogenize the tissue samples [29]. Protein samples were frozen immediately after extraction, in liquid nitrogen, and stored at -80 ^o^C. SDS-PAGE was performed with 10% gel, and western blotting was performed as described previously (29). Anti-leptin receptor polyclonal antibody (Novus Biologicals, NB120-5593, 1:2000 dilution) and anti-β-actin monoclonal antibody (Santa Cruz Biotechnology, sc-69879, 1:500 dilution) were used as 1^st^ antibodies and goat anti-rabbit IgG-HRP (Santa Cruz, sc-2004, 1:50000 dilution) and goat anti-mouse IgG-HRP (Santa Cruz, sc-2005, 1:50000 dilution) were used as 2^nd^ antibodies, respectively. Luminescence was detected using Immobilon Western Chemiluminescent HRP Substrate (Merck) and Image analyzer (ImageQuant LAS 4000 mini, GE Healthcare Life Sciences). Results were digitized with ImageQuant TL (GE Healthcare Life Sciences). All values were normalized to the mean value of 20C.

### Measurement of plasma leptin and sOb-R concentrations

Plasma leptin and plasma sOb-R concentrations were measured with Leptin ELISA Kit (Morinaga Institute of Biological Science, Inc.) and mouse Leptin R DuoSet ELISA (R&D Systems), respectively. The detection range for the ELISA kit was 0.2–12.8 ng/mL and the coefficient of variation was less than 10%, according to the manufacturer.

### Statistics

Data are expressed as means ± SEM. For differences between two groups, Student’s *t*-test or Welch’s *t*-test was performed for data with equal or unequal homogeneity of variance, respectively. Mann-Whitney *U* test was performed for non-normal data sets. For analyzing differences among three groups, one-way ANOVA and *post hoc* tests were used. The Kruskal–Wallis test was used for non-normally distributed data sets. Two-way ANOVA was performed to evaluate the effects of two factors simultaneously. *Post hoc* comparison was performed with Tukey-Kramer test when significant difference among groups was observed in Kruskal-Wallis test. All statistical analyses were performed using Statistics 2008 (Social Survey Research Information Co., Ltd., Tokyo, Japan) for Excel, and the differences were considered significant at *P* < 0.05.

## Results

### Effect of protein restriction and fasting/re-feeding on hepatic Ob-R mRNA in rats

**Fig 1:**
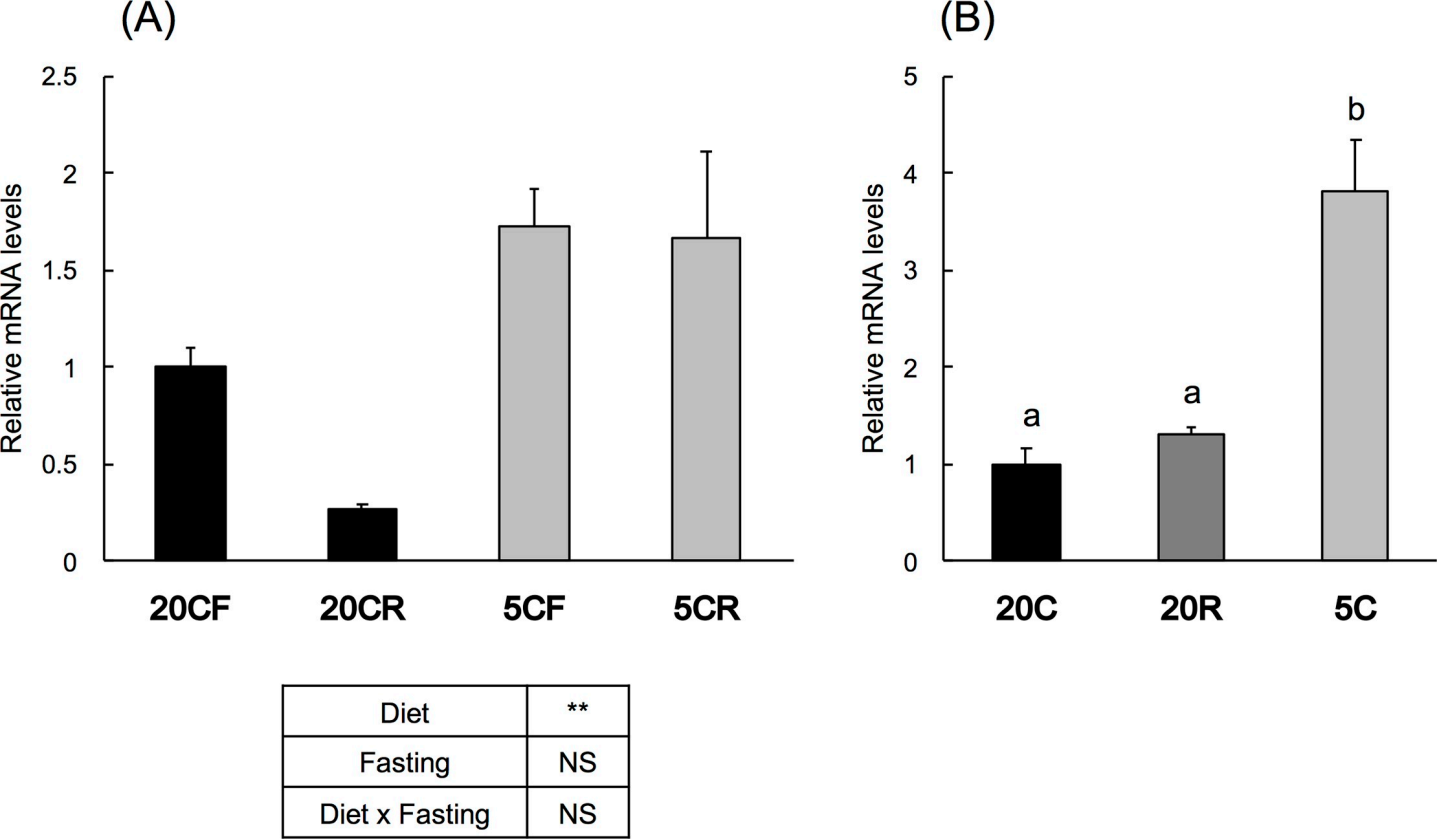
Effect of protein restriction on hepatic leptin receptor mRNA level in rats. (A) Rats were fed a control diet (20C) or a low-protein diet (5C) for 7 d and sacrificed after 12-h fasting (20CF, 5CF) or 12-h fasting followed by 12-h re-feeding (20CR, 5CR). Leptin receptor mRNA was measured by real-time PCR and results were expressed relative to that of 20CF, means ± standard errors (n = 5). Results of two-way ANOVA are given below the graph (NS, not significant; **, *p* < 0.01). (B) Rats were fed a control diet *ad libitum* (20C), fed a low-protein diet *ad libitum* (5C), or pair-fed a control diet with 5C (20R) for 16 d. Ob-R mRNA was measured by real-time PCR and expressed as means ± standard errors (n = 5). Results with different alphabet are statistically different (*P* < 0.05).

Average daily food intake of 20C was larger than that of 5C, and final body weight was higher in 20C-fed groups and lower in fasted groups (Table 4). Results of two-way ANOVA demonstrated that *Ob-R* mRNA level was increased by protein restriction, although not significantly influenced by fasting (Fig 1A).

**Table 4:** Body weight and food intake of rats and mice.

	Initial body weight (g)	Final body weight (g)	Food intake (g/day)
*Experiment with rats fed protein resticted diet in fasted or refed condition*			
20CF	175.4 ± 4.1	228.5 ± 6.5	20.3 ± 0.6
20CR	173.1 ± 2.5	241.0 ± 2.4	20.1 ± 0.4
5CF	175.1 ± 2.9	171.1 ± 1.9	16.5 ± 0.7
5CR	173.6 ± 2.3	186.3 ± 3.5	16.6 ± 0.6
Two-way ANOVA	NS	Diet, *P*<0.01; Fasting, *P*<0.01	Diet, *P*<0.01
*Experiment with rats under protein restriction or food restriction*			
20C	101.0 ± 1.2	231.9 ± 4.0a	21.0 ± 0.5a
20R	100.2 ± 1.1	164.4 ± 2.2b	12.7 ± 0.0b
5C	100.2 ± 0.8	101.6 ± 3.5c	12.8 ± 1.0b
*Experiment with mice fed diet protein restricted diet*			
20C	21.5 ± 0.2	23.3 ± 0.4	4.2 ± 0.3
5C	21.6 ± 0.4	22.2 ± 0.5	4.5 ± 0.2

Values are means±SEM (n = 5).

NS, not significant

Values with different alphabet were significantly different (*P*<0.05).

### Effect of protein restriction and food restriction on hepatic Ob-R mRNA in rats

Average food consumption was higher for 20C than for 20R and 5C, and final body weights were highest in 20C, followed by, in order, 20R and 5C (Table 4). Hepatic *Ob-R* mRNA levels increased 3.8-fold by protein restriction but were not influenced by food restriction (Fig 1B).

### Effect of protein restriction on Ob-R mRNA and protein levels in mice

**Fig 2:**
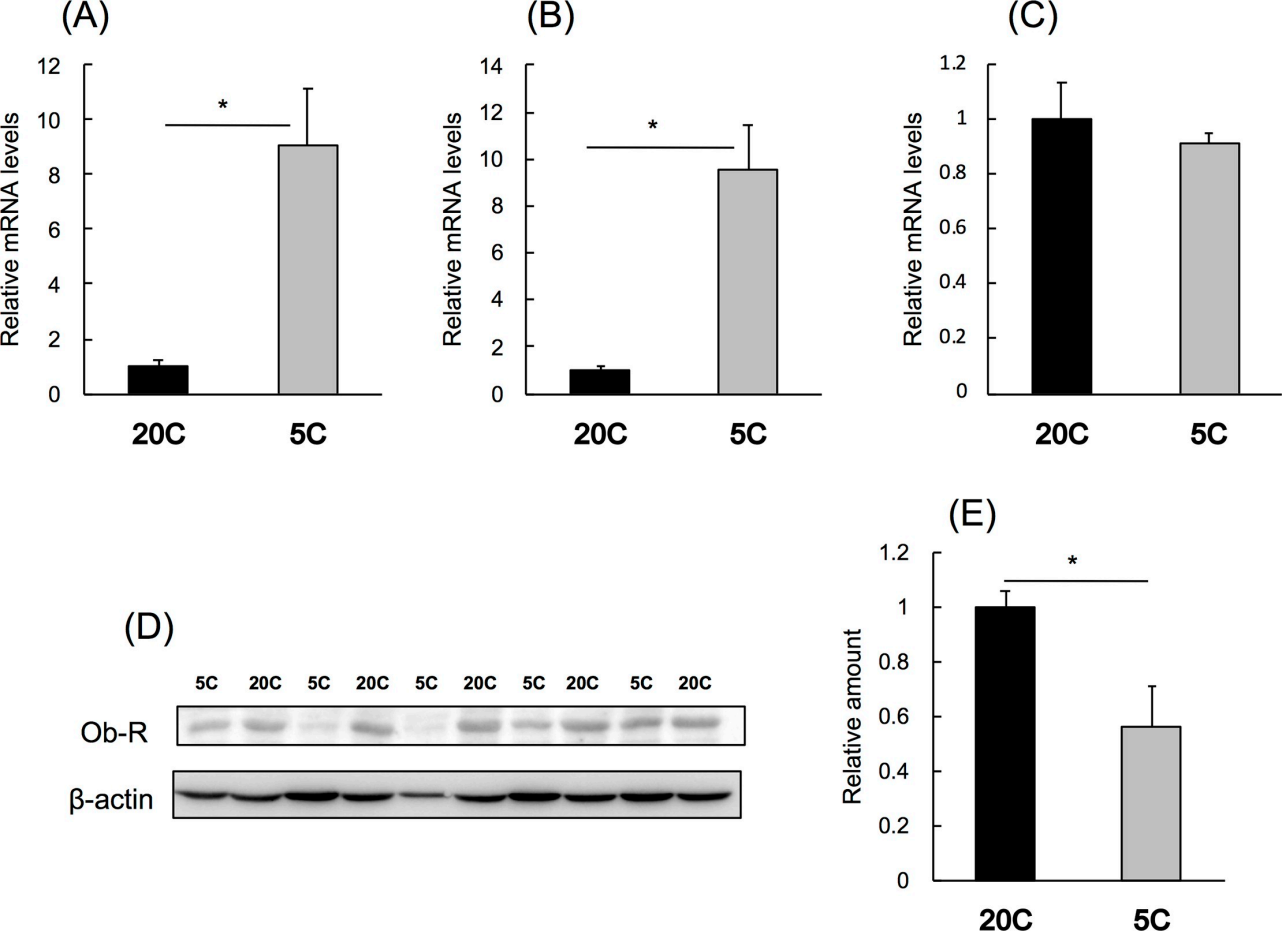
Effect of protein restriction on leptin receptor mRNA and protein levels in mice. C57BL/6 mice were fed 20C or 5C *ad libitum* for 7 d. *Ob-R* mRNA (A), trans-membrane region of *Ob-R* mRNA (B), and *Ob-R* mRNA in hypothalamus (C) was measured by real-time PCR. Hepatic *Ob-R* and *β-actin* were measured by western blotting. Images (D) and quantification results (E) are shown. Results are expressed relative to those of 20C, means ± standard errors (n = 5). Statistical difference between the groups are shown as *, *P* < 0.05.

Food intake and body weight were not affected by protein restriction (Table 4), while the rate of change in body weight over the 8-day period was higher for 20C than for 5C (20C, 0.90 ± 1.14%; 5C, -4.86 ± 1.04%; *P* < 0.01). Hepatic levels of *Ob-R* mRNA and the trans-membrane region of *Ob-R* increased 9.1- and 9.6-fold by protein restriction, respectively (Fig 2A and 2B), while *Ob-R* mRNA levels in the hypothalamus remained unchanged (Fig 2C). Hepatic Ob-R protein levels decreased by 44% upon protein restriction (Fig 2D and 2E).

### Effect of protein restriction on plasma leptin and sOb-R level in mice

**Fig 3:**
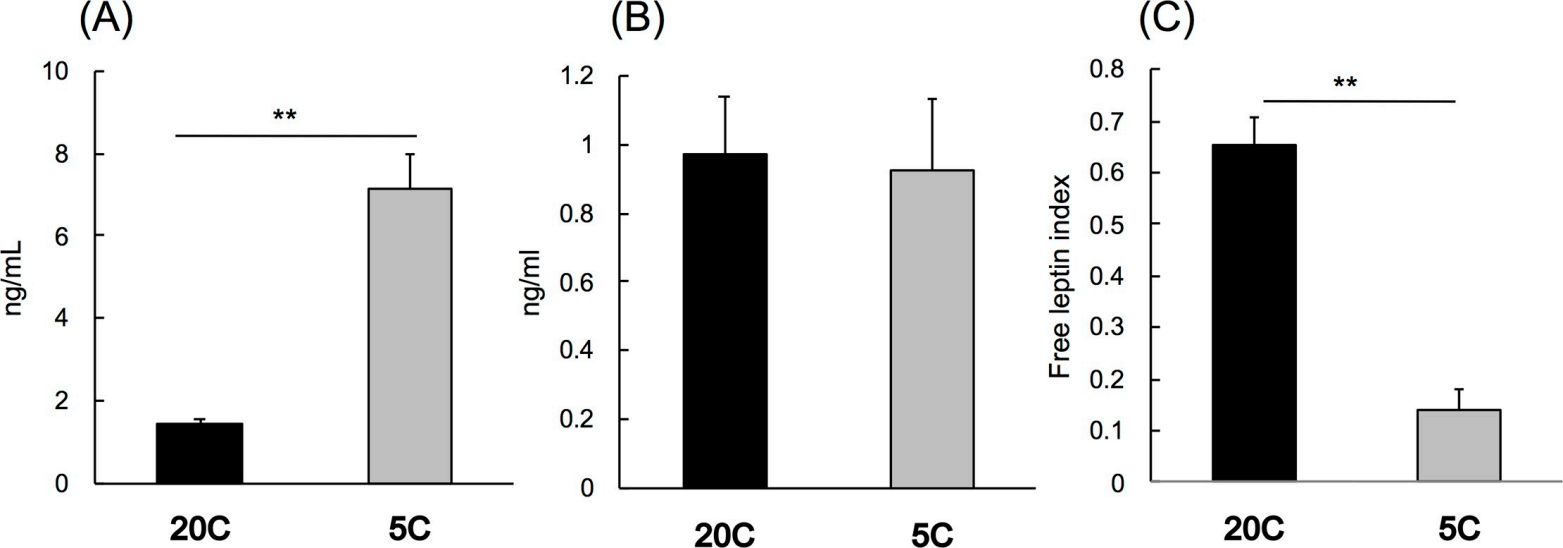
Effect of protein restriction on plasma leptin and leptin receptor in mice. C57BL/6 mice were fed 20C or 5C *ad libitum* for 7 d. Plasma sOb-R (A) and plasma leptin (B) concentrations were measured with ELISA. Free leptin index was calculated from plasma leptin and sOb-R (C). Results are expressed as means ± standard errors (n = 5). Statistical difference between the groups are shown as **, *P* < 0.01.

The plasma sOb-R concentration increased significantly (4.9-fold) by protein restriction (Fig 3A), while the plasma leptin concentration remained unchanged (Fig 3B). The free leptin index, calculated from plasma sOb-R and leptin concentrations, was significantly reduced (by 78.4%) by protein restriction (Fig 3C).

### Effect of amino acid deprivation on Ob-R mRNA in H4IIE cells

**Fig 4:**
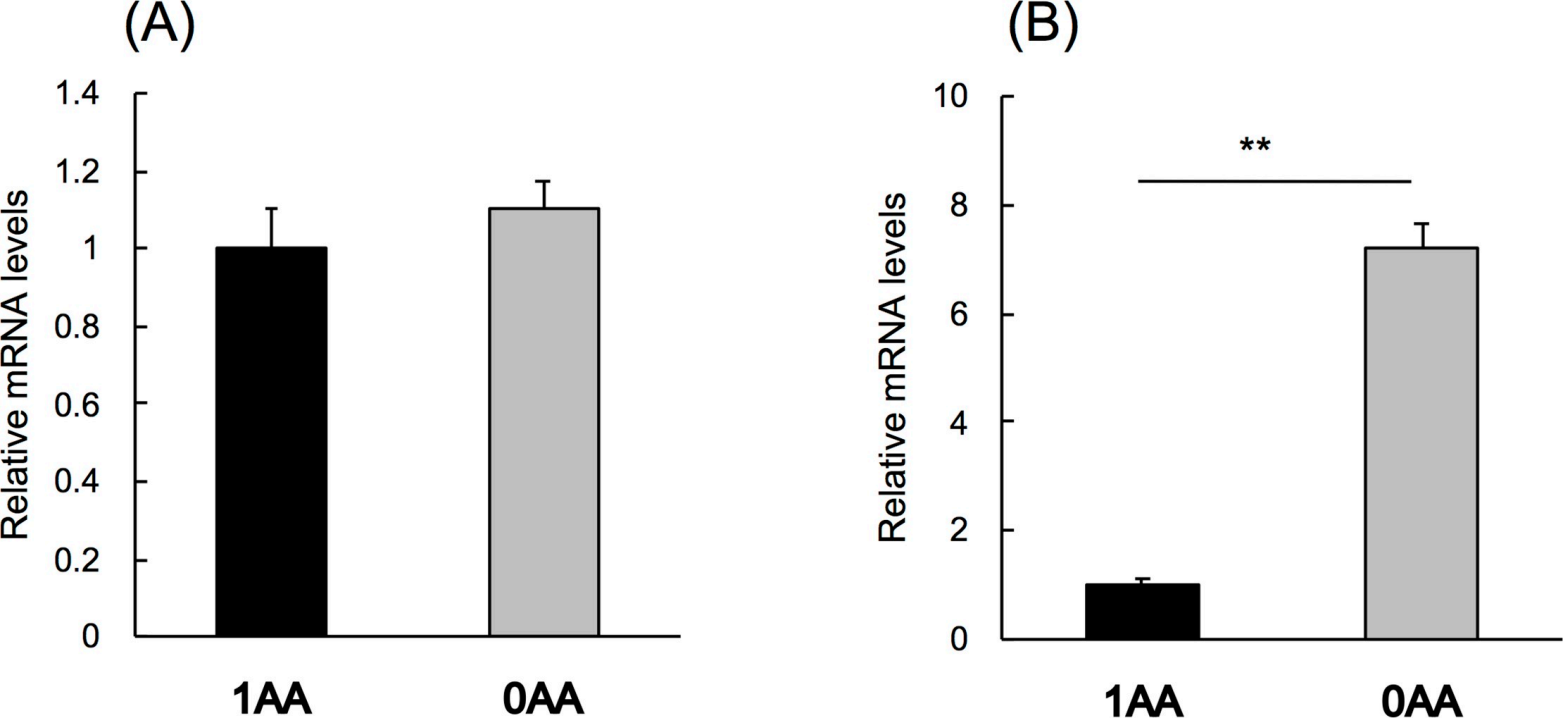
Effect of amino acid deprivation on Ob-R mRNA in H4IIE cells. H4IIE cells were cultured in media with amino acids (1AA) or without amino acids (0AA) for 6 h. *Ob-R* mRNA and *CHOP* mRNA were measured by real-time PCR. Results were expressed relative to those of 1AA, means ± standard errors (n = 5 or 6). Statistical difference between the group are shown as **, *P* < 0.01.

*Ob-R* mRNA in H4IIE cells was not affected by amino acid deprivation in the culture medium (Fig 4A). However, mRNA of *CHOP*, known as an amino acid-regulated gene, was significantly increased by amino acid deprivation (Fig 4B).

## Discussion

In the first half of this study, we investigated the regulation of *Ob-R* mRNA using rats as a commonly used animal model in nutritional research, and we used mice in the latter half since the specific sOb-R ELISA Kit was more available for mice than for rats. Results from this study clearly demonstrated that hepatic *Ob-R* mRNA expression was enhanced by low-protein diet in rats and mice. Twenty-four hours of fasting had earlier been reported to increase hepatic *Ob-R* mRNA levels [25]; however, our current results demonstrated that protein restriction was a stronger regulator of hepatic *Ob-R* mRNA than fasting. Furthermore, in our experiment, food consumption was reduced in protein-restricted rats, which might be a cause for the increase in *Ob-R* mRNA levels [25, 27, 30]. Therefore, effect of reduced food intake on *Ob-R* mRNA level was examined in another experiment, which demonstrated that dietary restriction alone did not enhance hepatic *Ob-R* mRNA expression. These results, together, showed that dietary protein restriction is a predominant regulator of hepatic *Ob-R* mRNA compared to fasting and food restriction.

We examined the effect of low-protein diet on Ob-R, precisely with mice. Hepatic *Ob-R* mRNA level was increased by low-protein diet in mice; however, hepatic Ob-R protein level was decreased. On the other hand, plasma sOb-R level was significantly increased by protein restriction. In addition, mRNA levels of *Ob-R* transmembrane domain increased because of protein restriction. The results demonstrated that production of membrane-anchored Ob-R was up-regulated in the liver, cleaved by protease and released into circulation as sOb-R. In this study, under fasting condition, levels of *Ob-R* mRNA in the hypothalamus did not increase due to protein deficiency; however, they were reported to be increased in another study [31]. Therefore, liver is probably the main source of plasma sOb-R in protein-restricted animals. Increased hepatic *Ob-R* mRNA and plasma sOb-R are common responses under conditions of protein restriction and fasting [25]. Increase in *Ob-R* mRNA levels in response to low protein diet was observed in female as well as male mice (S1 Fig).

Plasma sOb-R was reported to increase by restricted feeding and fasting [25, 27, 30], as well as by abnormal insulin signaling in the liver [26]. These results indicated that reduced hepatic insulin signaling might induce Ob-R expression. In rats fed low-protein diet, insulin secretion was low while tyrosine phosphorylation of insulin receptor substrate-2 was increased followed by enhanced hepatic insulin signaling in the liver [32]. Therefore, reduced insulin signaling may not be a cause for the increased production of sOb-R in protein-restricted animals. We investigated the direct effect of amino acid deprivation on *Ob-R* mRNA using H4IIE cells. *Ob-R* mRNA did not increase by amino acid deprivation whereas *CHOP* mRNA, which is known to be up-regulated by amino acid restriction through amino acid response element (AARE), increased [33]. These results demonstrated that *Ob-R* gene expression is not directly regulated by amino acids through AARE. As another possible regulatory mechanism, in protein restriction, we had earlier reported induction of fibroblast growth factor (FGF) 21 [28]. FGF21 was reported to up-regulate *Ob-R* mRNA in mouse liver through βKlotho/FGF receptor-1 [34]. However, induction of hepatic *Ob-R* mRNA by protein restriction was also observed in FGF21-knockout mice, to the same extent as in the wild-type mice (S1 Fig.), hence implying that increase of Ob-R occurred in FGF21-independent manner in protein-restricted mice. The precise mechanism to increase hepatic *Ob-R* mRNA by dietary protein restriction is unknown at present.

Plasma leptin concentration has been reported to either increase or decrease under low-protein diet, implicating leptin resistance in some cases [35–37]. In this study, plasma leptin concentration was not influenced by protein restriction and free leptin level was reduced. One possible physiological interpretation of this reduction by protein restriction may be to reduce leptin activity and maintain appetite in malnourished animals. sOb-R injection has been reported to block leptin action and increase food consumption in rats [38]. Although food consumption of the protein-restricted rats was reduced in this study, drastic reduction of food intake was possibly prevented by lowering the free leptin level. However, food intake was not reduced in protein-restricted mice in this study. A difference in the response to a low protein diet with respect to food consumption has been observed in various animal experiments using rats and mice [36]; therefore, role of leptin in the regulation of food intake under protein malnutrition is yet to be clarified.

Another possible physiological implication is that increased sOb-R influences energy expenditure. In our previous results, weight of white adipose tissue (WAT) was reduced and *uncoupling protein (UCP)-1* expression in WAT was increased in protein-restricted mice, which together suggested that energy expenditure in WAT was increased by protein restriction [28]. Increased energy expenditure in protein-restricted rats was also reported by other researchers [36]. We have clarified that plasma FGF21 concentration was induced in protein-restricted rats and mice [28], which may cause increased UCP1 expression and energy expenditure in adipose tissues [37]. Since leptin has been reported to increase expression of UCP-1 and -2 in brown and white adipose tissues [39], reduced leptin activity by sOb-R suppresses energy expenditure and exerts an effect opposite to that of FGF21. It is also possible that increased sOb-R may enhance leptin activity and work in cooperation with FGF21 on adipose tissue. Effect of sOb-R to reserve leptin and enhance its activity was demonstrated previously in mice over-expressing Ob-Re [20], which supports this possibility.

## Conclusions

In conclusion, low-protein diet increased hepatic *Ob-R* mRNA expression and plasma sOb-R concentration without increasing hepatic membrane-anchored Ob-R. Furthermore, we clarified that protein restriction is a predominant regulator of hepatic *Ob-R* mRNA compared to fasting and food restriction. Because plasma leptin level was not changed, free leptin level in plasma was significantly reduced by protein restriction, which may regulate leptin activity and influence appetite and energy expenditure. Induction of hepatic *Ob-R* mRNA and plasma sOb-R may contribute to a complex regulation of lipid metabolism under protein malnutrition.

## Supporting information

S1 Fig Effect of protein restriction on hepatic *Ob-R* mRNA in FGF21 knockout (KO) and wild type (WT) mice. Male (A) and female (B) mice were fed a control diet with 20% casein (20C) or a low protein diet (5C) for 10 days as reported previously [28]. Hepatic *Ob-R* mRNA was measured by realtime PCR and results were expressed as relative value to 20C-WT, means ± SEM (n = 5). Results of two-way ANOVA are given below the graph (NS, not significant; **, *P*<0.01).
(TIFF)

## References

[1] Zhang Y, Proenca R, Maffei M, Barone M, Leopold L, Friedman JM. Positional cloning of the mouse obese gene and its human homologue. Nature. 1994;372(6505):425–32. 10.1038/372425a0 . 7984236

[2] Frederich RC, Löllmann B, Hamann A, Napolitano-Rosen A, Kahn BB, Lowell BB, et al Expression of ob mRNA and its encoded protein in rodents. Impact of nutrition and obesity. J Clin Invest. 1995;96(3):1658–63. 10.1172/JCI118206 7657836 PMC185793

[3] Halaas JL, Gajiwala KS, Maffei M, Cohen SL, Chait BT, Rabinowitz D, et al Weight-reducing effects of the plasma protein encoded by the obese gene. Science. 1995;269(5223):543–6. 10.1126/science.7624777 . 7624777

[4] Pelleymounter MA, Cullen MJ, Baker MB, Hecht R, Winters D, Boone T, et al Effects of the obese gene product on body weight regulation in ob/ob mice. Science. 1995;269(5223):540–3. 10.1126/science.7624776 . 7624776

[5] Campfield LA, Smith FJ, Guisez Y, Devos R, Burn P. Recombinant mouse OB protein: evidence for a peripheral signal linking adiposity and central neural networks. Science. 1995;269(5223):546–9. 10.1126/science.7624778 . 7624778

[6] Levin N, Nelson C, Gurney A, Vandlen R, de Sauvage F. Decreased food intake does not completely account for adiposity reduction after ob protein infusion. Proc Natl Acad Sci U S A. 1996;93(4):1726–30. 10.1073/pnas.93.4.1726 8643697 PMC40010

[7] Tartaglia LA, Dembski M, Weng X, Deng N, Culpepper J, Devos R, et al Identification and expression cloning of a leptin receptor, OB-R. Cell. 1995;83(7):1263–71. 10.1016/0092-8674(95)90151-5 . 8548812

[8] Fei H, Okano HJ, Li C, Lee GH, Zhao C, Darnell R, et al Anatomic localization of alternatively spliced leptin receptors (Ob-R) in mouse brain and other tissues. Proc Natl Acad Sci U S A. 1997;94(13):7001–5. 10.1073/pnas.94.13.7001 9192681 PMC21274

[9] Wang MY, Zhou YT, Newgard CB, Unger RH. A novel leptin receptor isoform in rat. FEBS Lett. 1996;392(2):87–90. 10.1016/0014-5793(96)00790-9 . 8772180

[10] Myers MG, Cowley MA, Münzberg H. Mechanisms of leptin action and leptin resistance. Annu Rev Physiol. 2008;70:537–56. 10.1146/annurev.physiol.70.113006.100707 . 17937601

[11] Schaab M, Kausch H, Klammt J, Nowicki M, Anderegg U, Gebhardt R, et al Novel regulatory mechanisms for generation of the soluble leptin receptor: implications for leptin action. PLoS One. 2012;7(4):e34787 Epub 2012/04/24. 10.1371/journal.pone.0034787 22545089 PMC3335825

[12] Akasaka Y, Tsunoda M, Ogata T, Ide T, Murakami K. Direct evidence for leptin-induced lipid oxidation independent of long-form leptin receptor. Biochim Biophys Acta. 2010;1801(10):1115–22. Epub 2010/07/01. 10.1016/j.bbalip.2010.06.009 . 20601111

[13] Haynes WG, Morgan DA, Walsh SA, Mark AL, Sivitz WI. Receptor-mediated regional sympathetic nerve activation by leptin. J Clin Invest. 1997;100(2):270–8. 10.1172/JCI119532 9218503 PMC508189

[14] Ghilardi N, Ziegler S, Wiestner A, Stoffel R, Heim MH, Skoda RC. Defective STAT signaling by the leptin receptor in diabetic mice. Proc Natl Acad Sci U S A. 1996;93(13):6231–5. 10.1073/pnas.93.13.6231 8692797 PMC39004

[15] Margetic S, Gazzola C, Pegg GG, Hill RA. Leptin: a review of its peripheral actions and interactions. Int J Obes Relat Metab Disord. 2002;26(11):1407–33. 10.1038/sj.ijo.0802142 . 12439643

[16] Schaab M, Kratzsch J. The soluble leptin receptor. Best Pract Res Clin Endocrinol Metab. 2015;29(5):661–70. Epub 2015/09/06. 10.1016/j.beem.2015.08.002 . 26522452

[17] Lammert A, Kiess W, Bottner A, Glasow A, Kratzsch J. Soluble leptin receptor represents the main leptin binding activity in human blood. Biochem Biophys Res Commun. 2001;283(4):982–8. 10.1006/bbrc.2001.4885 . 11350082

[18] Yang G, Ge H, Boucher A, Yu X, Li C. Modulation of direct leptin signaling by soluble leptin receptor. Mol Endocrinol. 2004;18(6):1354–62. Epub 2004/03/11. 10.1210/me.2004-0027 . 15016839

[19] Yannakoulia M, Yiannakouris N, Blüher S, Matalas AL, Klimis-Zacas D, Mantzoros CS. Body fat mass and macronutrient intake in relation to circulating soluble leptin receptor, free leptin index, adiponectin, and resistin concentrations in healthy humans. J Clin Endocrinol Metab. 2003;88(4):1730–6. 10.1210/jc.2002-021604 . 12679465

[20] Lou PH, Yang G, Huang L, Cui Y, Pourbahrami T, Radda GK, et al Reduced body weight and increased energy expenditure in transgenic mice over-expressing soluble leptin receptor. PLoS One. 2010;5(7):e11669 Epub 2010/07/20. 10.1371/journal.pone.0011669 20652026 PMC2907393

[21] Ogier V, Ziegler O, Méjean L, Nicolas JP, Stricker-Krongrad A. Obesity is associated with decreasing levels of the circulating soluble leptin receptor in humans. Int J Obes Relat Metab Disord. 2002;26(4):496–503. . 12075576 10.1038/sj.ijo.0801951

[22] Stein K, Vasquez-Garibay E, Kratzsch J, Romero-Velarde E, Jahreis G. Influence of nutritional recovery on the leptin axis in severely malnourished children. J Clin Endocrinol Metab. 2006;91(3):1021–6. Epub 2005/12/13. 10.1210/jc.2005-1394 . 16352686

[23] Monteleone P, Fabrazzo M, Tortorella A, Fuschino A, Maj M. Opposite modifications in circulating leptin and soluble leptin receptor across the eating disorder spectrum. Mol Psychiatry. 2002;7(6):641–6. 10.1038/sj.mp.4001043 . 12140788

[24] Medici V, Ali MR, Seo S, Aoki CA, Rossaro L, Kim K, et al Increased soluble leptin receptor levels in morbidly obese patients with insulin resistance and nonalcoholic fatty liver disease. Obesity (Silver Spring). 2010;18(12):2268–73. Epub 2010/05/06. 10.1038/oby.2010.95 20448542 PMC4820322

[25] Cohen P, Yang G, Yu X, Soukas AA, Wolfish CS, Friedman JM, et al Induction of leptin receptor expression in the liver by leptin and food deprivation. J Biol Chem. 2005;280(11):10034–9. Epub 2005/01/11. 10.1074/jbc.M413684200 . 15644325

[26] Cohen SE, Kokkotou E, Biddinger SB, Kondo T, Gebhardt R, Kratzsch J, et al High circulating leptin receptors with normal leptin sensitivity in liver-specific insulin receptor knock-out (LIRKO) mice. J Biol Chem. 2007;282(32):23672–8. Epub 2007/06/07. 10.1074/jbc.M704053200 . 17556363

[27] Chan JL, Blüher S, Yiannakouris N, Suchard MA, Kratzsch J, Mantzoros CS. Regulation of circulating soluble leptin receptor levels by gender, adiposity, sex steroids, and leptin: observational and interventional studies in humans. Diabetes. 2002;51(7):2105–12. 10.2337/diabetes.51.7.2105 . 12086939

[28] Ozaki Y, Saito K, Nakazawa K, Konishi M, Itoh N, Hakuno F, et al Rapid increase in fibroblast growth factor 21 in protein malnutrition and its impact on growth and lipid metabolism. Br J Nutr. 2015;114(9):1410–8. Epub 2015/09/02. 10.1017/S0007114515002846 . 26330054

[29] Ozaki Y, Takeda T, Akanishi N, Hakuno F, Toyoshima Y, Takahashi S, et al Insulin injection restored increased insulin receptor substrate (IRS)-2 protein during short-term protein restriction but did not affect reduced insulin-like growth factor (IGF)-I mRNA or increased triglyceride accumulation in the liver of rats. Biosci Biotechnol Biochem. 2014;78(1):130–8. 10.1080/09168451.2014.877825 . 25036495

[30] Gallardo N, Arribas C, Villar M, Ros M, Carrascosa JM, Martínez C, et al ObRa and ObRe are differentially expressed in adipose tissue in aged food-restricted rats: effects on circulating soluble leptin receptor levels. Endocrinology. 2005;146(11):4934–42. Epub 2005/07/21. 10.1210/en.2005-0220 . 16037380

[31] Mitchell SE, Nogueiras R, Morris A, Tovar S, Grant C, Cruickshank M, et al Leptin receptor gene expression and number in the brain are regulated by leptin level and nutritional status. J Physiol. 2009;587(Pt 14):3573–85. Epub 2009/06/02. 10.1113/jphysiol.2009.173328 19491239 PMC2742282

[32] Toyoshima Y, Tokita R, Taguchi Y, Akiyama-Akanishi N, Takenaka A, Kato H, et al Tissue-specific effects of protein malnutrition on insulin signaling pathway and lipid accumulation in growing rats. Endocr J. 2014;61(5):499–512. Epub 2014/03/13. . 24621780 10.1507/endocrj.ej13-0514

[33] Bruhat A, Averous J, Carraro V, Zhong C, Reimold AM, Kilberg MS, et al Differences in the molecular mechanisms involved in the transcriptional activation of the CHOP and asparagine synthetase genes in response to amino acid deprivation or activation of the unfolded protein response. J Biol Chem. 2002;277(50):48107–14. Epub 2002/09/25. 10.1074/jbc.M206149200 . 12351626

[34] Adams AC, Cheng CC, Coskun T, Kharitonenkov A. FGF21 requires βklotho to act in vivo. PLoS One. 2012;7(11):e49977 Epub 2012/11/27. 10.1371/journal.pone.0049977 23209629 PMC3507945

[35] Du F, Higginbotham DA, White BD. Food intake, energy balance and serum leptin concentrations in rats fed low-protein diets. J Nutr. 2000;130(3):514–21. 10.1093/jn/130.3.514 . 10702578

[36] Aparecida de França S, Dos Santos MP, Garófalo MA, Navegantes LC, Kettelhut IoC, Lopes CF, et al Low protein diet changes the energetic balance and sympathetic activity in brown adipose tissue of growing rats. Nutrition. 2009;25(11–12):1186–92. Epub 2009/06/17. 10.1016/j.nut.2009.03.011 . 19535223

[37] Pezeshki A, Zapata RC, Singh A, Yee NJ, Chelikani PK. Low protein diets produce divergent effects on energy balance. Sci Rep. 2016;6:25145 Epub 2016/04/28. 10.1038/srep25145 27122299 PMC4848496

[38] Zhang J, Scarpace PJ. The soluble leptin receptor neutralizes leptin-mediated STAT3 signalling and anorexic responses in vivo. Br J Pharmacol. 2009;158(2):475–82. Epub 2009/05/06. 10.1111/j.1476-5381.2009.00246.x 19422379 PMC2757686

[39] Commins SP, Watson PM, Padgett MA, Dudley A, Argyropoulos G, Gettys TW. Induction of uncoupling protein expression in brown and white adipose tissue by leptin. Endocrinology. 1999;140(1):292–300. 10.1210/endo.140.1.6399 . 9886838

