# Supplementary figures and images for: Dietary protein restriction increases hepatic leptin receptor mRNA and plasma soluble leptin receptor in male rodents
Source: PLoS One. 2019 Jul 15;14(7):e0219603. doi: 10.1371/journal.pone.0219603 (PMC6629078; doi:10.1371/journal.pone.0219603)

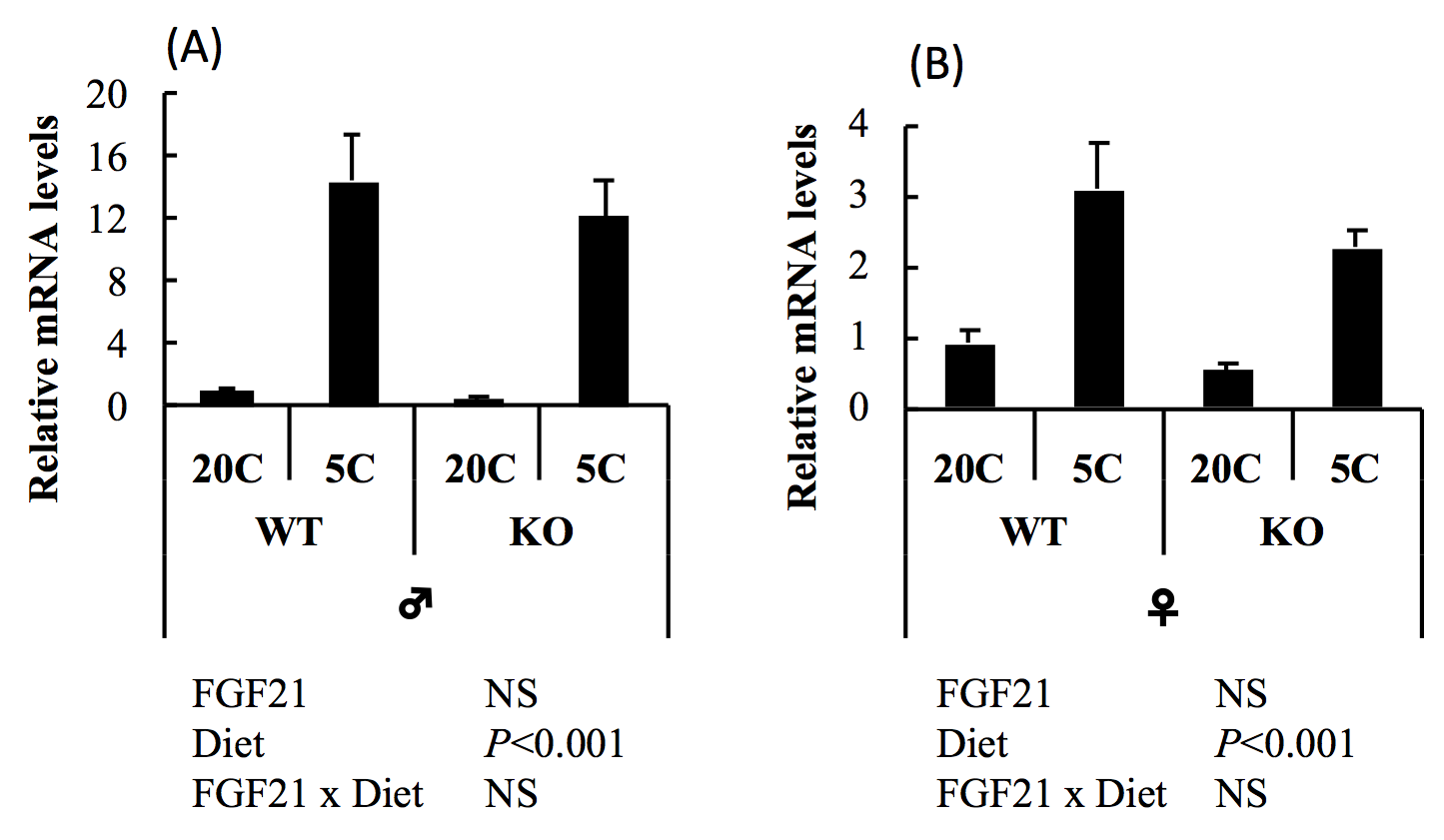

Supplement: S1 Fig — Male (A) and female (B) mice were fed a control diet with 20% casein (20C) or a low protein diet (5C) for 10 days as reported previously [28]. Hepatic Ob-R mRNA was measured by realtime PCR and results were expressed as relative value to 20C-WT, means ± SEM (n = 5). Results of two-way ANOVA are given below the graph (NS, not significant; **, P<0.01). (TIFF) [file pone.0219603.s001.tiff]
